# *Mycobacterium tuberculosis* VII secretion system effector molecule Rv2347c blocks the maturation of phagosomes and activates the STING/TBK1 signaling pathway to inhibit cell autophagy

**DOI:** 10.1128/spectrum.01188-24

**Published:** 2024-09-23

**Authors:** Zhiyong Jiang, Junfeng Zhen, Yuerigu Abulikena, Chaoyun Gao, Lingxi Huang, Tingting Huang, Jianping Xie

**Affiliations:** 1 Institute of Modern Biopharmaceuticals, State Key Laboratory Breeding Base of Eco-Environment and Bio-Resource of the Three Gorges Area, Key Laboratory of Eco-Environments in Three Gorges Reservoir Region, Ministry of Education, School of Life Sciences, Southwest University, Chongqing, China; Shenzhen University School of Medicine, Shenzhen, China

**Keywords:** phagolysosome, autophagy, STING

## Abstract

The VII secretion system is the main channel for *Mycobacterium tuberculosis* (*MTB*) to secrete virulence proteins. The ESAT-like proteins EsxA/B and EsxW/V in the RD region of its genome have been used as targets for vaccine antigens. However, the function of EsxO/P has not been explored, although it was predicted to potentially induce Th1 cell responses as a vaccine development target. In this study, the VII secretion system effector molecule Rv2347c was heterologously expressed in *Mycobacterium smegmatis* and found to inhibit the expression of the early marker RAB5 of phagosomes, thus preventing the maturation process of phagosomes toward lysosomes, and activated the host cytoplasmic sensing pathway. It inhibited autophagy and activated IFNβ transcription through the STING/TBK1 pathway promoting the host’s survival. Therefore, Rv2347c plays an important role in the pathogenesis of *MTB* with the potential to be utilized as a new target for tuberculosis vaccine development.

**IMPORTANCE:**

We found that the ESAT-like protein Rv2347c (EsxP) can inhibit the maturation of phagosomes, leading to mycobacterium escape from phagosomes into the cytoplasm, which triggers the host’s cytoplasmic sensing pathway STING/TBK1, inhibiting autophagy and upregulating IFNβ transcription, which contributes to the survival of mycobacterium in the host cell. We also found that Rv2347c was able to activate host immunity by activating NF-κB via STING and promoting the transcription of downstream pro-inflammatory factors. Meanwhile, the host also produces IL-1β to repair phagosome maturation arrest via the STING-mediated non-NF-κB pathway.

## INTRODUCTION

Tuberculosis is an infectious disease caused by a single pathogen, *Mycobacterium tuberculosis* (*MTB*). As a country with a high burden of tuberculosis, China ranks third in the world, with an incidence rate of 52/100,000, second only to India and Indonesia (1). *MTB* is an intracellular pathogen that has evolved multiple strategies to evade the host immune system with secreted virulence proteins playing a crucial role. The ESX secretion system of *MTB* plays a significant role in the secretion of virulence proteins (2).

*MTB* has five ESX systems, and ESX-1 can help mycobacteria to escape from phagocytic vesicles in host cells. *MTB* and many pathogenic mycobacteria can even express complete recombinant strains of *esx-1* BCG::RD1, which can be transferred from phagocytes of infected macrophages or dendritic cells to cytoplasm, while strains with *esx-1* deficiency are confined in vesicles and less toxic than wild type, which may be the result of the interaction between ESAT-6 and biofilm (3). ESX-2 can assist the transport of *MTB* exotoxin CpnT into host cells and promote the toxic effect of CpnT (4). EsxH, the substrate of ESX-3 subtype, inhibits the transport of ESCRT, blocks the presentation of MHC-II antigen, and inhibits the activation of CD4+ T cells. EsxH can limit the ability of effector CD4+ T cells to recognize infected macrophages and eliminate *MTB* (5). ESX-4 can affect the rearrangement of the actin cytoskeleton and thus the phagocytosis of macrophages. Knocking out *eccD4* can improve the secretion of ESX-1 and ESX-5 substrates, which reflects the evolutionary relationship of the ESX system (6). ESX-5 exists in slow-growing mycobacteria, which is related to nutrient intake and secretion of immune-modified proteins (7).

Currently, EsxA/EsxB are the most extensively studied substrates for ESX-1 secretion. EsxA/EsxB play an important role in the infection of host phagocytes by *MTB*, which can rupture the phagosome membrane transferring *MTB* from the phagosome to the host cytoplasm and promote the formation of granulomas. Knockout of ESX-1 strains of *MTB* and *Mycobacterium marinum* leads to loss of its hemolytic activity (8). EsxA can also mediate the process of the host cell glycolysis pathway shifting toward ketone synthesis, inducing lipid droplet accumulation in cells, thereby promoting the persistence of mycobacteria (9). In addition, EsxA also plays an important role in the host DNA cytoplasmic sensing pathway by acting on the cytoplasmic DNA sensor IFI204/IFI16, activating the STING/TBK1 pathway, and upregulating Interferon-β (IFNβ) expression ultimately promoting bacterium survival (10, 11). Twenty-three ESAT-like proteins have been discovered, among which the more specific are EsxA/B, EsxV/W, and EsxO/P, which exist in RD1, RD7, and RD9, respectively (the RD region is the differential region between the genomes of *MTB* and *Bacillus Calmette Guerin* (*BCG*) (12). The *esxA* gene fragment can be supplemented in the recombinant BCG strain, and the EsxA protein can be secreted into the extracellular space through the Esx-5 system. It only enhances the immunogenicity of the vaccine and does not increase its reactogenicity (13). EsxV can activate host T cells’ memory response and reduce bacterium loading in animals infected with *MTB*. On this basis, the ID93/GLA-SE vaccine was developed for adult vaccination, which has entered phase II clinical trials, indicating that T7SSs-related molecules in the RD region have the potential to serve as new targets for tuberculosis vaccines (12). However, there is limited research on the function of EsxO/P in the RD7 region. Currently, most reports on EsxO/P are based on bioinformatics and crystal structure analysis predictions, as well as its ability to upregulate IFNγ and TNFα expression in phagocytes, which triggers Th1 immune response, suggesting its potential as a new vaccine target, but the functional mechanism has not been elucidated (14, 15). Only one article mentions that EsxO can weaken the host immune defense by inducing host cell death and genomic instability, but the mechanism is not yet clear (16). EsxP, EsxW, EsxM, EsxJ, and EsxK belong to the QILSS family (all of them contain glutamine, isoleucine, leucine, and two serine in C-terminal) and their amino acid sequences are highly conserved, with only a few amino acid differences, and they are all predicted as CD8+ T cell epitopes (17). Although some immunogenic epitopes are located in the region with 100% sequence homology, some immunodominant epitopes are also found in the protein sequence diversity region of the QILSS subfamily. Even the difference of a single residue in epitope sequence changes the response frequency to these antigens. For example, the mutation of T58, a key amino acid residue in the QILSS subfamily, causes the change in T cell recognition effect, which indicates that different members of the QILSS family will have different immune responses to the host (18). Therefore, more research is needed to explore the function of EsxO/P and its mechanism of action in the host, which is also beneficial for the development of new tuberculosis vaccines.

As an intracellular pathogen, *MTB* enters the phagosome after being engulfed by macrophages. In order to escape from the antibacterial environment of acidified lysosomes, *MTB* blocks a series of phagosome maturation events that occur after phagocytosis (19). Many secreted proteins are involved in this process, such as SapM, EsxA, and PtPA (20–22). Here, we found that the ESAT-like protein Rv2347c (EsxP) can inhibit the maturation of phagosomes, leading to mycobacterium escape from phagosomes into the cytoplasm, which triggers the host cytoplasmic sensing pathway STING/TBK1, inhibiting autophagy and upregulating IFNβ transcription, which contributes to the survival of mycobacterium in the host. We also found that Rv2347c was able to activate host immunity by activating NF-κB via STING and promoting the transcription of downstream pro-inflammatory factors. Meanwhile, the host also produces Interleukin-1β (IL-1β) to repair phagosome maturation arrest via the STING-mediated non-NF-κB pathway.

## RESULTS

### Rv2347c promotes the survival of recombinant mycobacterium in host cells

**Fig 1:**
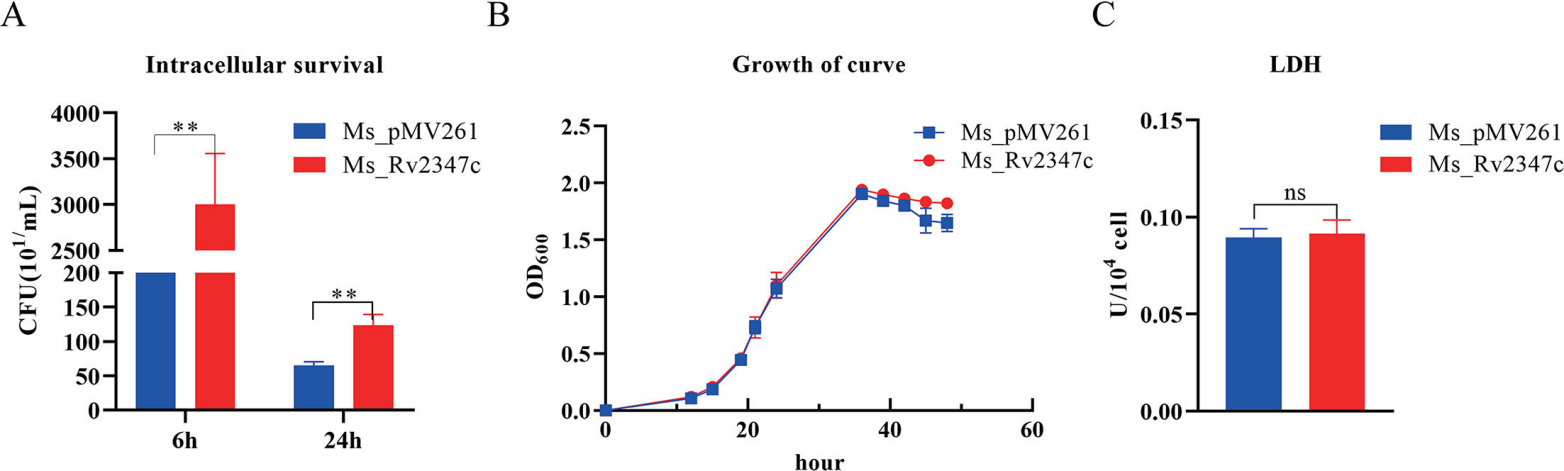
Rv2347c enhances the viability of bacteria in the host. (A) Loading bacteria quantity determination. THP-1 cells were infected with Ms_Rv2347c and Ms_pMV261, and treated with 0.025% SDS for 10 minutes at 6 hours and 24 hours, respectively, to detect the survival of bacteria in the host. (B) The growth of Ms_Rv2347c and Ms_pMV261 in 7H9 medium was measured at OD_600_ every 3 hours. (C) Analyzing the activity of LDH in THP-1 cell culture filtrate infected with Ms_Rv2347c and Ms_pMV261. All experiments were repeated at least three times. Two-tailed unpaired Student’s *t*-test was used for statistical analysis, significance: *P < 0.05, **P < 0.01, ns for no difference.

Rv2347c, as a virulence factor of the ESX system, may be related to the survival of bacteria in the host. THP-1 cells were infected by Ms_Rv2347c and Ms_pMV261, and the survival of mycobacterium in cells at 6 hours and 24 hours was detected. We show that Rv2347c can enhance the survival ability of bacteria in the host. It was found that the amount of loading bacteria was significantly higher at 6 hours as well as 24 hours in the Ms_Rv2347c infected group compared to the Ms_pMV261 group (Fig. 1A). At the same time, we measured the growth of the two strains and found that there was no difference in the growth rate between the two strains, ruling out the effect of differences in the growth of the strains themselves (Fig. 1B), suggesting that Rv2347c enhances bacterial survival in the host. Multiple reasons exist for the increased viability of bacteria in the host. Does Rv2347c, as a virulence factor, promote bacterial survival by enhancing bacterial virulence to host cells, causing cell death? Cell death will lead to the leakage of lactate dehydrogenase (LDH) into cell culture filtrate, so the content of lactate dehydrogenase in culture filtrate can reflect the amount of cell death. We assayed the culture filtrate for lactate dehydrogenase content. The results showed that there was no difference in lactate dehydrogenase between Ms_Rv2347c and Ms_pMV261 infected cell culture filtrate (Fig. 1C), which indicated that Rv2347c would not aggravate cell death.

### Rv2347c is beneficial to the escape of bacteria from lysosomes

**Fig 2:**
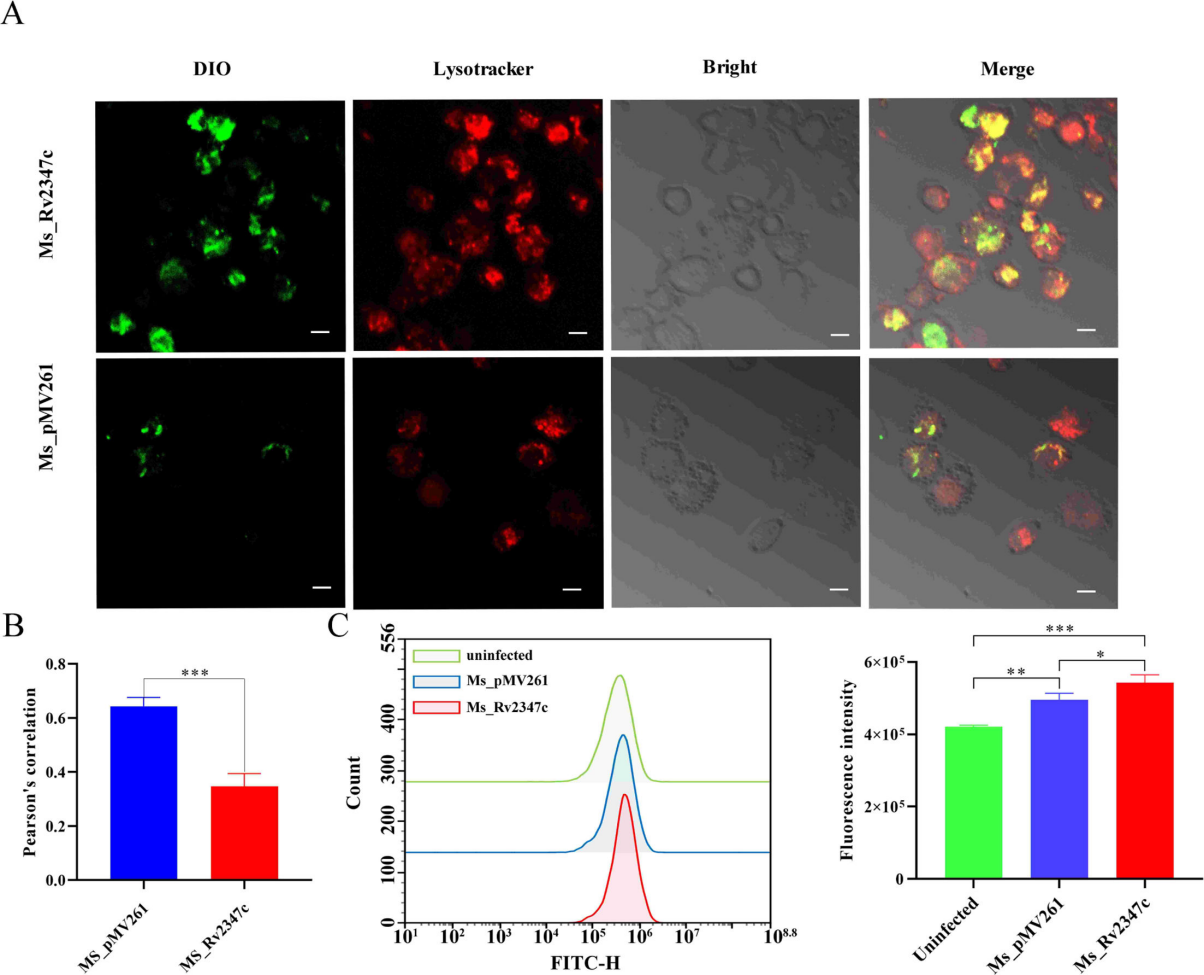
Rv2347c ruptures the integrity of the lysosomal membrane and mitochondrial membrane. (A) The fluorescence localization of bacteria and lysosomes observed by confocal microscope. Bacteria were dyed green with Dio, lysosomes were dyed red, and the fluorescence intensity curve was drawn by ImageJ. The scale bars depict 5 µm. (B) Pearson’s correlation coefficient of (A). (C) The infected macrophages were stained with rhodamine 123, the mitochondrial membrane potential was measured by flow cytometry, and the results were analyzed by 10,000 gated cells. All experiments were repeated at least three times. Two-tailed unpaired Student’s *t*-test was used for statistical analysis, significance: *P < 0.05, **P < 0.01, ***P < 0.001.

The ability of *MTB* to evade and adapt to the host immune system is critical as a successful intracellular pathogen. It is reported that *MTB* mainly escapes into the cytoplasm by rupturing the lysosomal membrane in order to facilitate its own survival (21). Rv2347c is highly expressed under hypoxic and low pH conditions (23), whereas in the host environment, *MTB* faces hypoxic and low pH conditions in lysosomes after phagocytosis by macrophages, indicating that Rv2347c may be beneficial for bacteria to resist the acidic environment of lysosomes. We examined the co-location of bacteria with lysosomes by fluorescence after 24 hours in infection of THP-1 cells, and to observe whether Rv2347c affects the escape of bacteria from lysosomes. Lysotracker dyed lysosomes red, and Dio dyed mycobacteria green. The results showed that the overlapping area between the Ms_pMV261 strain and lysosomes was significantly higher than that of the Ms_Rv2347c strain (Fig. 2A and B). This shows that Rv2347c can promote the escape of bacteria in lysosomes. Bacterial escape from lysosomes leads to leakage of lysosomal contents, which compromises mitochondrial membrane integrity and ultimately affects the mitochondrial membrane potential. Furthermore, we measured the mitochondrial membrane potential using rhodamine 123. As a cationic dye, it is able to enter into the mitochondria through the mitochondrial plasmonic kinetic potential. When the mitochondrial membrane potential is impaired, it leads to rhodamine 123 leakage, which leads to the determination of the mitochondrial membrane potential by fluorescence intensity. The results show that the fluorescence intensity of the Ms_Rv2347c infected group is significantly higher than Ms_pMV261 group, and the fluorescence intensity of the Ms_pMV261 group is higher than the uninfected group. It suggested that mycobacterial infection led to the decrease of mitochondrial membrane potential, while Rv2347c further reduced the mitochondrial membrane potential, indicating that the integrity of the mitochondrial membrane was damaged (Fig. 2C). Based on the above evidence, Rv2347c can rupture the phagosome membrane and promote the escape of mycobacteria from lysosomes.

### Rv2347c inhibits the expression of early markers of phagosomes

**Fig 3:**
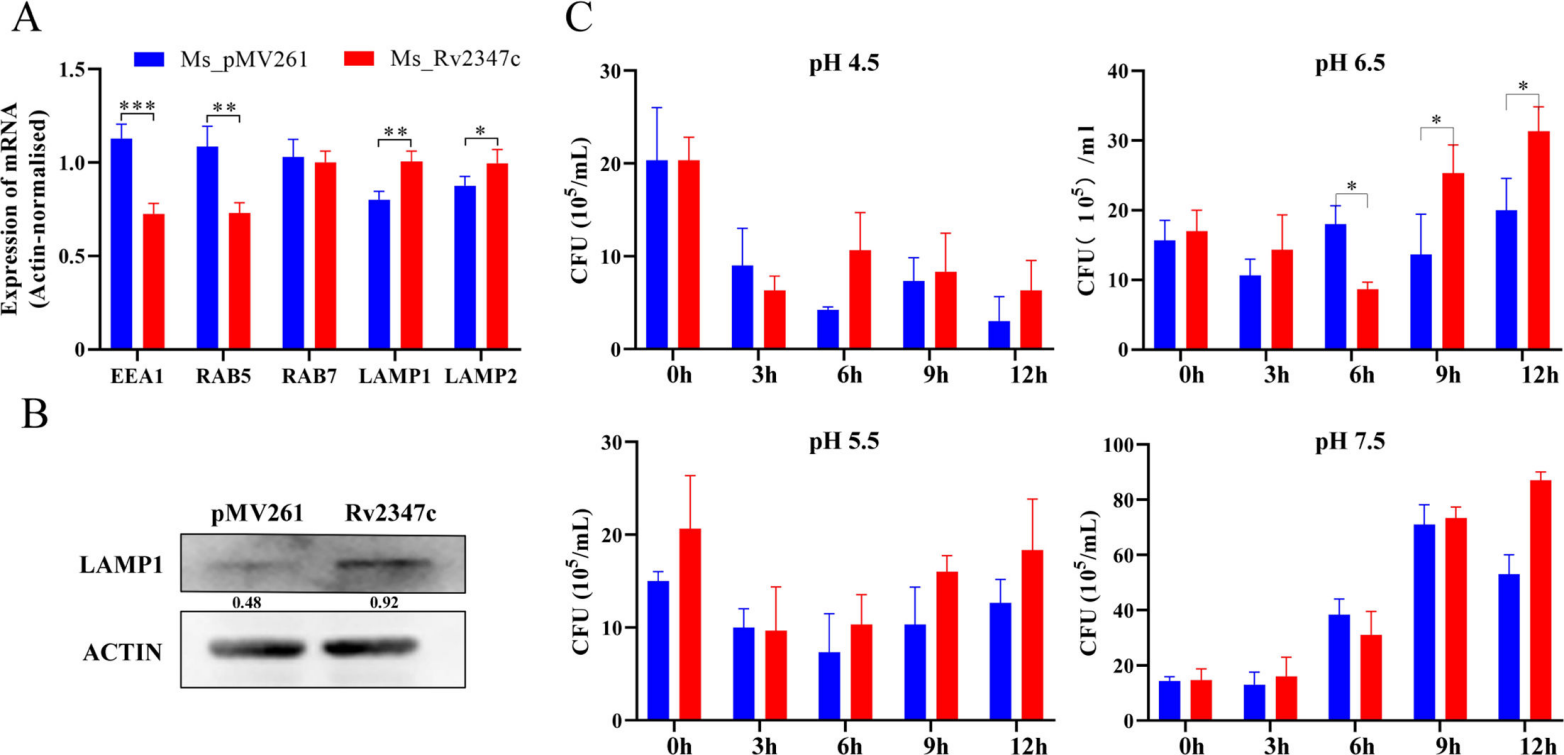
Expression levels of phagosomes’ markers in different periods. (A) RT-qPCR was used to detect the mRNA levels of markers of phagosomes in different periods when THP-1 was infected for 24 hours. (B) Western blot was used to detect the expression level of LAMP1 protein when THP-1 was infected for 24 hours. pMV261 refers to THP-1 cells infected with Ms_pMV261, while Rv2347c refers to THP-1 cells infected with Ms_Rv2347c. (C) Determination of the viability of mycobacteria at different pH levels. The colony formation units of overexpressed and empty vector strains were determined at 0, 3, 6, 9, and 12 hours at pH levels 4.5, 5.5, 6.5, and 7.5, respectively. All experiments were repeated at least three times. Two-tailed unpaired Student’s *t*-test was used for statistical analysis, significance: *P < 0.05, **P < 0.01, ***P < 0.001.

To investigate how Rv2347c disrupts the phagosome membrane, we examined the transcript levels of markers for each period of phagosome by reverse transcription-quantitative polymerase chain reaction (RT-qPCR) in different strains of bacteria after infecting the host for 24 hours. The results showed that compared with Ms_pMV261 infected group, the transcription levels of early phagosome markers *RAB5* and *EEA1* decreased, while the transcription levels of phagolysosome markers *LAMP1* and *LAMP2* increased, and there was no difference in the transcription level of late phagosome marker *RAB7* (Fig. 3A). Consistent with the transcriptional level, the Western blot results further demonstrated at the protein level that the expression level of LAMP1 was significantly higher in the Ms_Rv2347c infected group than in the Ms_pMV261 group (Fig. 3B). It shows that Rv2347c can inhibit the maturation of phagosome in the early stage, but it is beneficial to the transition from late stage of phagosome to lysosomal stage. In addition, we also simulated the intracellular pH environment of phagosome in different periods and analyzed the viability of bacteria. The results showed that under pH 6.5 treatment conditions, no difference in growth was observed at the initial 3 hours, and at 6 hours, the Ms_pMV261 strain was more viable than the Ms_Rv2347c strain. However, the viability was reversed at 9 hours versus 12 hours. No differences in the survival levels of the two strains were observed under pH 7.5 versus pH 5.5 treatment conditions. Furthermore, under treatment conditions at pH 4.5, neither bacterium was able to grow (Fig. 3C). Taken together, Rv2347c inhibits phagosome maturation by suppressing early phagosome expression of RAB5, leading to bacterial escape from the lysosome into the cytoplasm and facilitating its own survival.

### Rv2347c inhibits autophagy

**Fig 4:**
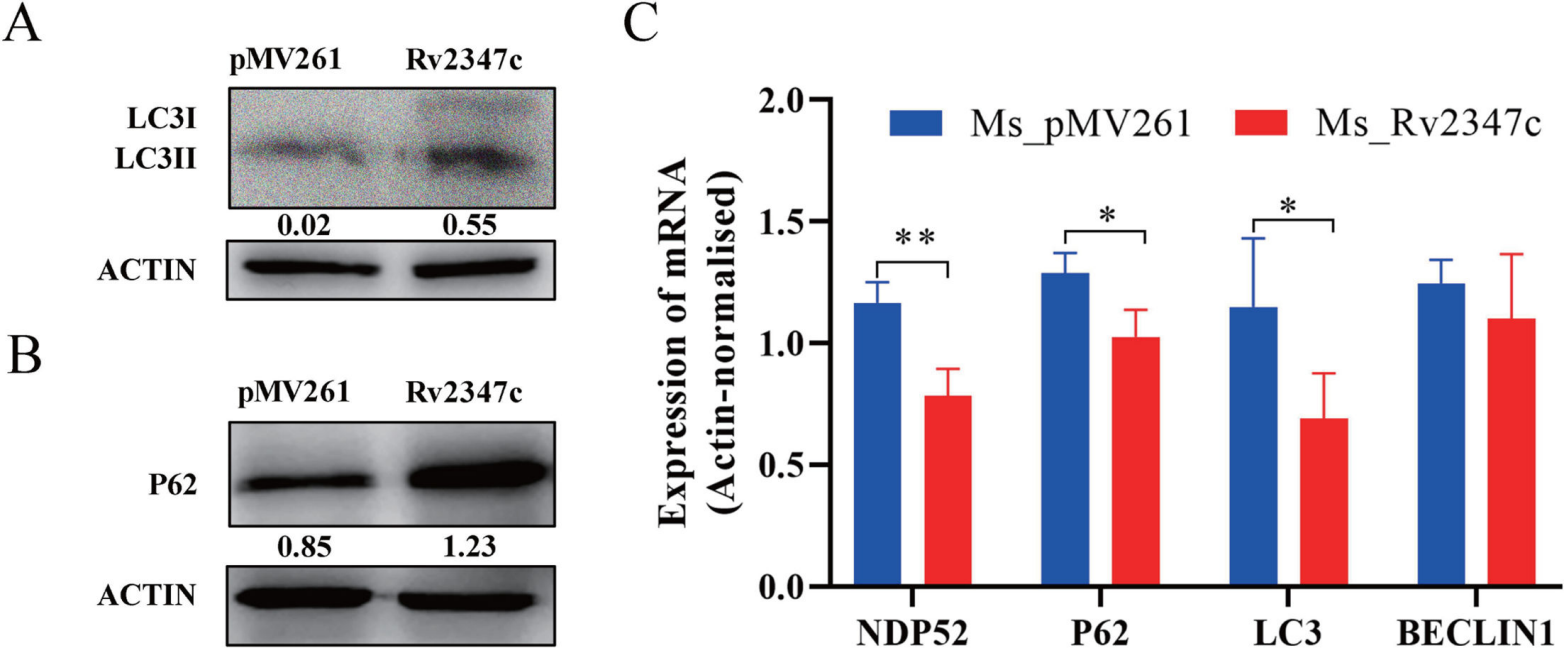
Expression of autophagy proteins LC3 II and P62 in Ms_Rv2347c and Ms_pMV261 infection. (A and B) After 24 hours of infection with Ms_Rv2347c and Ms_pMV261, the expression levels of LC3 protein and P62 protein of THP-1 were detected by Western blot. (C) RT-qPCR was used to detect the mRNA levels of NDP52, P62, LC3, and BECLIN1 when THP-1 was infected for 24 hours. pMV261 refers to THP-1 cells infected with Ms_pMV261, while Rv2347c refers to THP-1 cells infected with Ms_Rv2347c. All experiments were repeated at least three times. Two-tailed unpaired Student’s *t*-test was used for statistical analysis, significance: *P < 0.05, **P < 0.01.

The maturation of phagosome is closely related to autophagy. Autophagy requires the fusion of autophagosomes and phagolysosomes, and then the substances in autophagosomes are degraded. Therefore, we speculate that Rv2347c may be related to autophagy. To test this conjecture, we examined the accumulation of autophagy substrate P62 and the transformation of LC3 I to LC3 II in different strains after 24 hours of host infection by Western blot. The results showed that the content of P62 protein in Ms_Rv2347c infected group was significantly higher than the Ms_pMV261 group, and the ratio of LC3 I/LC3 II increased (Fig. 4A and B), indicating that Rv2347c can inhibit the transformation from LC3 I to LC3 II, suggesting that Rv2347c can inhibit autophagy. We also measured the mRNA levels of autophagy-related proteins *NDP52*, *P62*, *BECLIN1*, and *LC3*. The results showed that compared with Ms_pMV261 infection group, the expression levels of *NDP52*, *P62*, and *LC3* mRNA in Ms_Rv2347c infection group decreased significantly, but there was no difference in the expression of *BECLIN1* (Fig. 4C). *BECLIN1* is related to autophagosome assembly, indicating that Rv2347c may not inhibit autophagy by affecting autophagosomes assembly.

### Rv2347c inhibits autophagy by activating STING/TBK1 pathway

**Fig 5:**
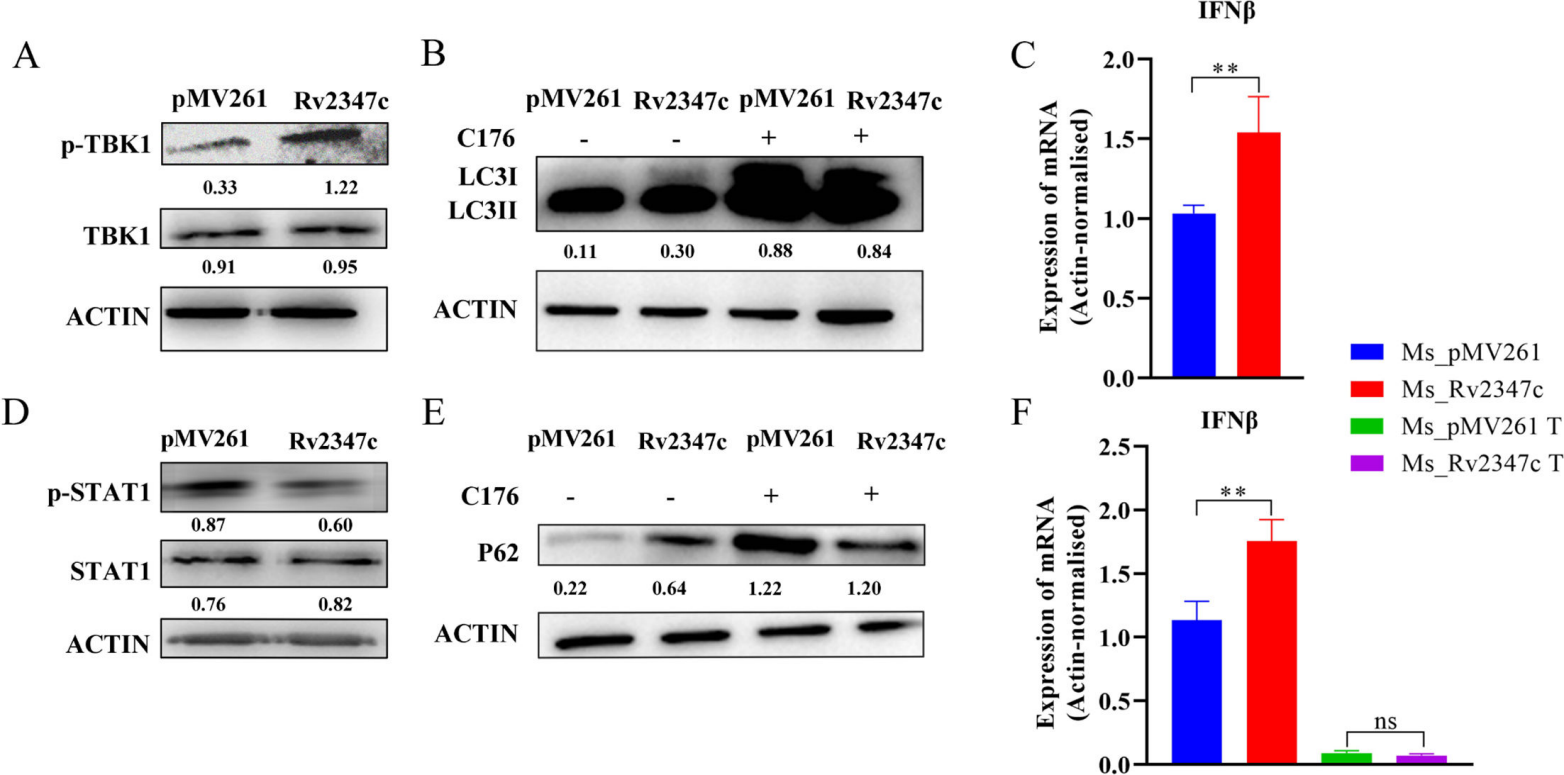
Rv2347c inhibits autophagy by promoting phosphorylation of TBK1. (A) After Ms_Rv2347c and Ms_pMV261 were infected with THP-1 for 24 hours, the TBK1 protein and its phosphorylation level of THP-1 were detected by Western blot. (B) Western blot was used to detect STAT1 and its phosphorylation level. (C) RT-qPCR was used to detect the transcription level of IFNβ of THP-1. (D) Ms_Rv2347c and Ms_pMV261 infected THP-1 cells treated with C176 or untreated, and the transcription level of IFNβ of THP-1 was detected by RT-qPCR. (E) Western blot was used to detect the expression of LC3 protein. (F) Western blot was used to detect the expression of P62 protein. pMV261 refers to THP-1 cells infected with Ms_pMV261, while Rv2347c refers to THP-1 cells infected with Ms_Rv2347c. All experiments were repeated at least three times. Two-tailed unpaired Student’s *t*-test was used for statistical analysis, significance: *P < 0.05, **P < 0.01, ns for no difference.

When bacteria escape from lysosomes to the host cytoplasm, they will be recognized by sensors in the cytoplasm and activate the downstream immune response. STING, as the adapter of sensor, plays an important role in the cytoplasmic sensing pathway. It has been found that *MTB* infection can stimulate STING and activate the recruitment of TBK1 and phosphorylation, thus activating the downstream IFNβ expression and promoting the survival of bacteria (11). Rv2347c is helpful for bacteria to enter the host cytoplasm, and it also plays a similar role in inhibiting the cytoplasmic response pathway. We analyzed the phosphorylation level of TBK1 after THP-1 infected the two strains, and found that the protein level of p-TBK1 in the Ms_Rv2347c infected group was significantly higher than the Ms_pMV261 group, but there was no difference in the total protein expression level of TBK1 (Fig. 5A). Then we measured the expression level of IFNβ by RT-qPCR and found that the transcription level of IFNβ in Ms_Rv2347c infection group increased (Fig. 5C). To clarify that Rv2347c acts through the STING/TBK1 pathway, we treated THP-1 cells with C-176, an inhibitor of the STING pathway, and examined the mRNA levels of IFNβ. The transcript levels of IFNβ in hosts infected by Ms_Rv2347c and Ms_pMV261 were found to be significantly lower in the inhibitor-treated than in the untreated group, while the inhibitor treatment eliminated the difference between the two strains (Fig. 5F). The results showed that Rv2347c could activate STING/TBK1 pathway and promote the expression of IFNβ. IFNβ can activate JAK/STAT1 pathway and promote the transcription of downstream antibacterial and antiviral-related genes. In order to explore the influence of the increase of IFNβ expression on the downstream pathway, we also analyzed the phosphorylation level of transcription factor STAT1 and found that Rv2347c inhibited the phosphorylation of STAT1 (Fig. 5D), thus inhibiting the transcription of downstream genes. Contrary to the previous results, Rv2347c may have other mechanisms to affect the activation of STAT1, which may be the reason why mycobacteria can still promote the survival of bacteria after upregulating IFNβ and may be related to IFNAR receptors (24).

IFNβ inhibits IFNγ-mediated autophagy (25). Does Rv2347c promote IFNβ production via STING/TBK1 and thus inhibit autophagy? In order to explore the relationship between STING and autophagy, we treated THP-1 cells infected with overexpressed and empty strains with STING inhibitor C176 and collected intracellular proteins at 24 hours, and measured autophagy proteins by Western blot. The results showed that the process of LC3 I to LC3 II conversion was further inhibited in the Ms_Rv2347c-infected and Ms_pMV261-infected groups after C176 treatment, but the ratios of LC3 I to LC3 II were much closer between the two groups, eliminating the inhibitory effect caused by Rv2347c (Fig. 5B). Furthermore, we also found that C176 treatment resulted in the accumulation of P62 and also eliminated the Rv2347c-induced accumulation of P62 protein (Fig. 5E), indicating that Rv2347c can inhibit autophagy by activating STING/TKB1 pathway. Taken together, the evidence suggests that Rv2347c can inhibit autophagy in host cells by promoting the phosphorylation of TBK1.

### Rv2347c activates NF-κB pathway through STING to enhance host immune response

**Fig 6:**
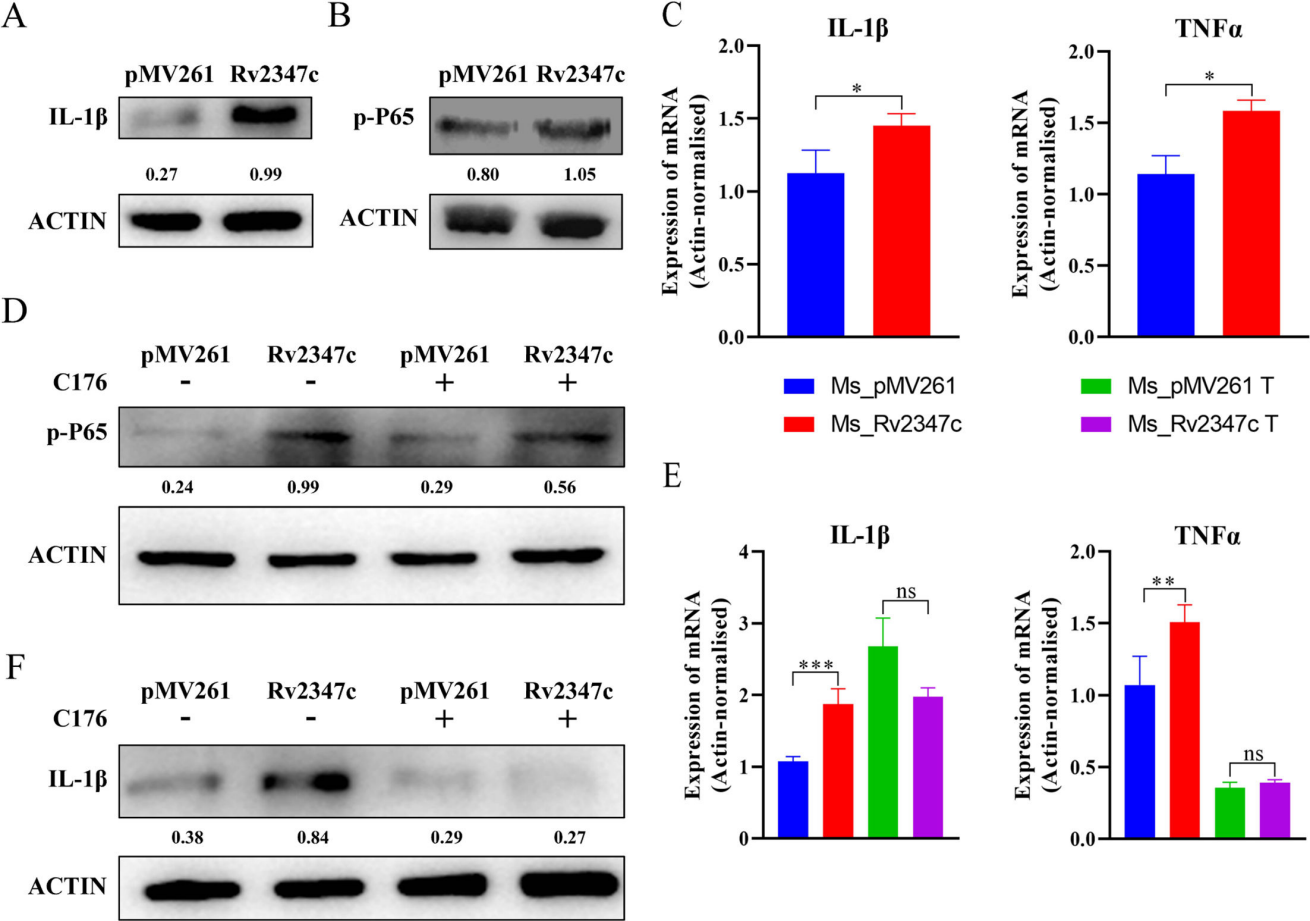
Rv2347c enhances the expression of inflammatory factors in the host by activating the NF-κB pathway through STING. Ms_Rv2347c and Ms_pMV261 were infected with THP-1 for 24 hours. (A) The protein level of IL-1β of THP-1 was detected by Western blot. (B) Western blot was used to detect the protein level of p-P65 of THP-1. (C) The transcription levels of IL-1β and TNFα of THP-1 were detected by RT-qPCR. (D) THP-1 cells treated with C176 or untreated with MS_ Rv2347c and Ms_pMV261 were infected, and the protein level of p-P65 of THP-1 was detected by Western blot. (E) RT-qPCR was used to detect the transcription levels of IL-1β and TNFα of THP-1. (F) Western blot was used to detect the protein level of IL-1β of THP-1. pMV261 refers to THP-1 cells infected with Ms_pMV261, while Rv2347c refers to THP-1 cells infected with Ms_Rv2347c. All experiments were repeated at least three times. Two-tailed unpaired Student’s *t*-test was used for statistical analysis, significance: *P < 0.05, **P < 0.01, ***P < 0.001, ns for no difference.

In order to overcome the influence of bacteria on phagosome maturation, the host can help phagocytes surmount the stagnation of phagosome maturation by secreting IL-1β (26). Whether Rv2347c can prevent the maturation of phagosome will also inhibit the expression of IL-1β, thus preventing the host from repairing the maturation of phagosome. We analyzed the transcription level of IL-1β and found that Rv2347c upregulated the expression of IL-1β, and also analyzed the protein level of IL-1β by Western blot. The results were consistent with RT-qPCR, suggesting that Rv2347c promotes IL-1β production to help the host repair the bacterial-induced stagnation of phagosome maturation (27), which may be related to the increased transcription levels of *LAMP1* and *LAMP2* (Fig. 6A and C).

How does Rv2347c promote the expression of IL-1β? It has been reported that IL-1β production and maturation are associated with NF-κB, and the STING pathway also affects NF-κB activation (28, 29). We aim to determine whether Rv2347c can upregulate IL-1β expression through STING/NF-κB pathway. To test this speculation, we analyzed the activation level of the NF-κB pathway and found that the phosphorylation level of P65 in the Ms_Rv2347c infection group increased significantly (Fig. 6B). We also measured the transcription level of TNFα, the downstream gene of NF-κB, and found that the transcription level of TNFα in Ms_Rv2347c infection group was significantly increased, suggesting that Rv2347c enhanced the activity of NF-κB and promoted the transcription of downstream genes, which was consistent with the upregulation of IL-1β expression (Fig. 6C). To ensure that IL-1β upregulation is achieved by activation of the STING/NF-κB pathway by Rv2347c, we treated THP-1 cells with C176. It was observed that the activation level of NF-κB in the Ms_Rv2347c infected group was significantly inhibited, but it was still higher than the Ms_pMV261 group, and the transcription of downstream genes was also significantly inhibited. However, the protein expression level of IL-1β in Ms_Rv2347c-infected and Ms_pMV261-infected groups under C176 treatment was significantly suppressed but the transcript level was significantly upregulated, and the difference caused by Rv2347c on IL-1β was eliminated at the same time, indicating that the translation of IL-1β was impeded, which suggests that Rv2347c does not affect IL-1β expression through the STING/NF-κB pathway to affect IL-1β expression, but is associated with IL-1β translation (Fig. 6D through F).

In summary, when bacteria enter the cytoplasm, Rv2347c can enhance the activity of P65 of NF-κB through the STING pathway, promote the transcription of downstream inflammatory factors, and activate the host immune response. In addition, Rv2347c also upregulates IL-1β expression through other pathways, and the STING pathway plays an important role in this.

## DISCUSSION

**Fig 7:**
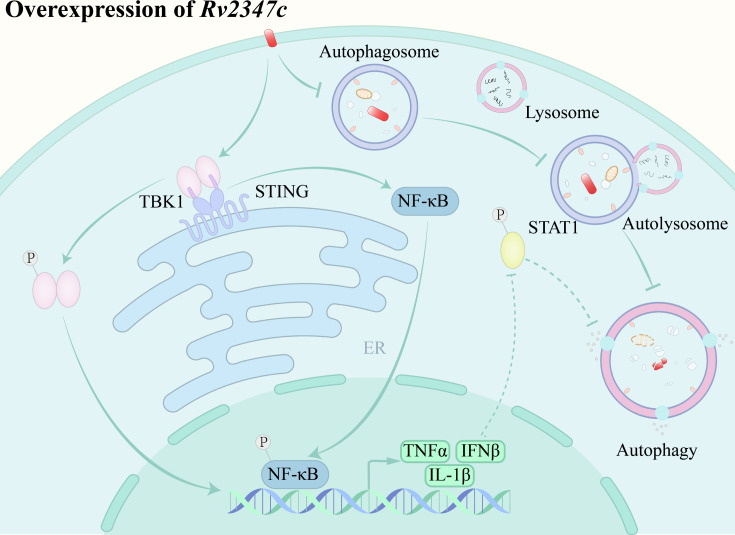
Pattern diagram of interaction mechanism between Rv2347c and host. Rv2347c can inhibit autophagy by inhibiting phagosome maturation. After entering the cytoplasm, IFNβ transcription is upregulated by activating STING and phosphorylating TBK1, which activates the host antibacterial immunity and promotes the survival of mycobacteria in the host cell. In addition, Rv2347c can also activate the transcription of downstream inflammatory factors through STING/NF-κB.

*MTB*, as a successful intracellular pathogen, is capable of evading the host immune through a variety of strategies, one of the more important steps being the escape from the acidic environment of the phagolysosome, a process that is achieved by influencing the maturation of the phagolysosome (30). It was previously widely believed that mycobacteria secrete EsxA through the ESX-1 system and thereby disrupt phagosome membranes, but Lienard et al. found that ESX-1-mediated phagosomes membrane rupture is not achieved by EsxA (31, 32). In the study, we found that the ESAT-like protein EsxP is also able to block phagosome maturation, leading to bacterial escape into the cytoplasm to evade killing by the acidic environment of the phagosomes. It also causes leakage of phagosome contents and damage to the integrity of the mitochondrial membrane, functions that are similar to previous parts of EsxA (Fig. 7 ). EsxP may be a previously unrecognized effector molecule of membrane lysis, or it may interact and act synergistically with ESX-1 system substrates. In addition, we found that EsxP blocked phagosome maturation by inhibiting the expression of *RAB5* and *EEA1*, and thus phagosome maturation for the same period that *MTB* was found to inhibit phagosome maturation in a previous study (33). It is hypothesized that EsxP may act by blocking vesicle fusion during periods of phagosomal RAB5 and RAB7 control.

Autophagy is a conserved process in eukaryotic evolution in which cytoplasmic components are surrounded and segregated by membranous structures and subsequently fused to lysosomes for degradation, while autophagy also plays an important role in innate defense against intracellular pathogen invasion (34, 35). Autophagy is an effective way for the host to limit *MTB* infection, although it has been found that *MTB* inhibits autophagy through the ESX-1 system substrate EspB as well as other secreted proteins SecA2, SapM (20, 25). However, it remains to be determined whether there are other secreted effector molecules involved in inhibiting this process of cellular autophagy. In this paper, we found that overexpression of Rv2347c resulted in the stagnation of phagosome maturation and the inability to form phagolysosomes, and it was hypothesized that overexpression of Rv2347c could inhibit the fusion of mycobacterium and autophagosomes, thereby inhibiting cellular autophagy and promoting the survival of mycobacterium in the host (Fig. 7). We examined the transcriptional and translational levels of autophagy markers LC3 and P62 after infection of THP-1 cells with Ms_Rv2347c by RT-qPCR and Western blot experiments, confirming that overexpression of Rv2347c inhibited cellular autophagy and favored bacterial survival in THP-1 cells. However, overexpression of Rv2347c did not show any difference in the transcriptional level of *BECLIN1* compared with the null group, indicating that Rv2347c inhibits autophagy mainly by inhibiting the maturation of phagolysosomes, which is one of the strategies of *MTB* to inhibit the immune defense of the host cells (36).

When *Mycobacterium* escapes to the cytoplasm, it is recognized by the host cytoplasmic sensing pathway, and the cytoplasmic sensor transmits the signal through STING, which in turn removes the bacteria through autophagy and activates IFNβ expression (10, 11). Although IFNβ plays an important role in the antiviral pathway, it promotes the survival of *MTB* in its infection, probably because of its ability to inhibit the antimycobacterial effect produced by IFNγ and enhance mycobacterial virulence (37, 38). Our data show that Rv2347c activates the STING/TBK1 pathway to promote IFNβ transcription and that autophagy levels in Rv2347c-infected THP-1 cells were further reduced after treatment with the STING inhibitor C176, suggesting that Rv2347c can affect autophagy through STING/TBK1 (Fig. 7). However, the specific cytoplasmic sensors that Rv2347c acts on are unclear and may be related to the known targets of *MTB* upon entry into the cytoplasm, namely cGAS, IFI204, and RNF144, a member of the RBR E3 ubiquitin ligase family (10, 11, 39). IFNβ binds to IFNAR to transmit the signal to STAT1 and phosphorylate it, for this reason, we analyzed STAT1, a transcription factor activated downstream of IFNβ and found that its activity was inversely inhibited, and STAT1 also acted as a transcription factor downstream of IFNγ, suggesting that IFNγ-induced effects were also inhibited, which may explain the negative regulation of IFNγ by IFNβ to inhibit the antimicrobial effect of IFNγ (40). Inhibition of STAT1 phosphorylation by Rv2347c might be related to IFNAR, and knockdown of IFNAR enhances the survival of susceptible mice in response to infection with hypervirulent strains HN878 and H37Rv. However, treatment with the influenza A virus as an inducer of IFNβ also reduces host clearance of *MTB*, and this discrepancy disappears in IFNAR−/− mice (41–43). Therefore, we hypothesized that Rv2347c may downregulate STAT1 phosphorylation levels by affecting IFNβ binding to IFNAR.

IL-1β plays an important role in host clearance of *MTB* infections, and one of the more important functions is to overcome the arrest of phagosome maturation, which is functionally antagonized by Rv2347c (27). We found that Rv2347c did not prevent IL-1β expression and maturation, which may be linked to the fact that Rv2347c inhibits early phagosome maturation but leads to upregulation of LAMP1 in the phagolysosomes phase. In addition, Rv2374c-mediated IL-1β production is consistent with NF-κB activation, and it has also been demonstrated that *MTB* can inhibit IL-1β production by affecting NF-κB (28, 44, 45). However, after the activation of NF-κB was de-activated by the inhibitor STING, the transcriptional level of IL-1β was increased despite the decrease in the production of IL-1β, suggesting that the promotion of IL-1β production by Rv2347c is not achieved through STING-activated NF-κB, but rather by some other unknown mechanism.

The ability of bacteria to intracellular survival and be transcriptionally active after being internalized requires maximal induction of IL-1β and IFNβ production (46). In this study, we found that Rv2347c was able to upregulate IFNβ through STING/TBK1, but the mechanism of STING activation by Rv2347c is not clear, and these effects were mainly related to phagosome rupture. Although Rv2347c was able to affect phagosome maturation by influencing early phagosome markers expression of *RAB5* and *EEA1*, how does it affect the phagosome maturation process, and whether it is related to the production of PI4P (47)? The ability of Rv2347c to promote IL-1β production and whether activation of upstream NLRP3 also affects pyroptosis in the host. An in-depth understanding of the role of Rv2347c in the host provides new insights into the pathogenesis of *MTB* and a theoretical basis for the development of EsxP as a new target for tuberculosis vaccine development.

## MATERIALS AND METHODS

### Bacteria and cell culture

*Mycobacterium smegmatis* mc^2^155 was cultured in Middlebrook 7H9 liquid medium containing 0.2% glycerol as a carbon source and 0.05% Tween-80 as an anticoagulant, or in Middlebrook 7H10 solid medium with 2% glycerol added. *Escherichia coli* DH5α was cultivated in Luria-Bertani medium. THP-1 was cultured in RPMI1640 medium which contain 10% fetal bovine serum.

### Recombinant bacterial construction

Amplify the coding region of Rv2347c from the genomic DNA of *Mycobacterium tuberculosis* and add six histidine residues at the C-terminus, then clone it into the pMV261 plasmid to produce pMV261-Rv2347c. Then, the pMV261-Rv2347c and pMV261 plasmids were electroporated into wild-type *Mycobacterium smegmatis* to produce Ms_Rv2347c and Ms_ pMV261 strains, respectively.

### Survival experiments of bacteria with different pH values

Different strains were cultured overnight in Middlebrook 7H9 liquid medium containing 0.2% glycerol as a carbon source and 0.05% Tween-80. The culture was collected, washed, and treated with 1× phosphate buffered saline (PBS) resuspended to OD_600_ = 0.8 and packaged in 7H9 medium with pH 4.5, 5.5, 6.5, and 7.5.

### Intracellular survival

THP-1 cells were inoculated in a 12-well plate at a density of 1 × 10^6^ with 0.1 µg/mL phorbol 12-myristate 13-acetate (PMA) and cultured for 2 days. Different strains infected cells with 10:1 multiplicity of infection (MOI). Four hours after the cells were infected, the culture medium was taken out, and the cells were washed with precooled 1× PBS and replaced with a new culture medium. After 6 hours and 24 hours of infection, the cells were washed three times and lysed with sterilized 0.025% (wt/vol) SDS in water. The lysed THP-1 cells were spotted on Middlebrook 7H10 plate with 10 times dilution, and the colonies were counted after 3–4 days at 37°C.

### RT-qPCR

Different strains infected cells with 10:1 MOI. Four hours after the cells were infected, the culture medium was taken out, and the cells were washed with precooled 1× PBS and replaced with new culture medium. After 24 hours of infection, the cells were washed three times, the cells were lysed with mRNA lysate, and RNA was extracted by RNA extraction kit (Promega), then the RNA was transformed into cDNA by reverse transcription kit (TAKARA), and RT-qPCR was performed by CFX96 real-time quantitative PCR instrument (BIO-RAD).

### Western blot

Different strains infected cells with 10:1 MOI. After 24 hours of infection, the cells were washed three times, and the cells were lysed with RIPA lysate to collect proteins. The protein was electrophoresed in 15% or 18% SDS-PAGE, and then transferred to nitrocellulose membrane (NC membrane) at a voltage of 15 V. The protein was sealed in a shaking at room temperature with 5% bovine serum albumin (BSA) for 2 hours, and then LC3, P62, TBK1, p-TBK1, STAT1, p-STAT1, IL-1β, LAMP1, ACTIN, and p-P65 were used. It was washed with Tris-Borate-Sodium Tween-20 (TBST) for five times, then incubated with corresponding goat anti-mouse or goat anti-rabbit antibodies for 2 hours at room temperature, and finally developed with ECL chemiluminescence kit.

### Mitochondrial membrane potential detection

Different strains infected cells with 10:1 MOI. After 24 hours of infection, Rh123 with a final concentration of 50 µg/mL was added and incubated at 37°C for 30 minutes for staining, and the fluorescence was detected by flow cytometry in fluorescein isothiocyanate (FITC) channel.

### Lysosomal acidification

Different strains infected cells with 10:1 MOI. After 24 hours of infection, the cells were washed three times, then 1 mL of Lysotracker-red diluent was added and incubated at 37°C for 30 minutes. The diluent was then removed, 1 mL of culture medium was added, and observed with a fluorescence confocal microscope.

### Determination of lactate dehydrogenase content

Different strains infected cells with 10:1 MOI. Four hours after the cells were infected, the culture medium was taken out, and the cells were washed with precooled 1× PBS and replaced with new culture medium. After 24 hours of infection, cell culture filtrate was collected and detected by lactate dehydrogenase detection kit (Solarbio).

### Statistics and analysis

GraphPad Prism software is used to perform statistical analysis on data (GraphPad). Double tailed unpaired Student’s *t*-test is used for statistical analysis, and unless otherwise specified, all results represent at least two independent biological experiments. Significance is as follows: *P < 0.05, **P < 0.01, and ***P < 0.001.
